# Link between sterile inflammation and cardiovascular diseases: Focus on cGAS-STING pathway in the pathogenesis and therapeutic prospect

**DOI:** 10.3389/fcvm.2022.965726

**Published:** 2022-08-22

**Authors:** Yao Du, Hui Zhang, Xiaoyan Nie, Yajun Qi, Shi Shi, Yingying Han, Wenchen Zhou, Chaoyong He, Lintao Wang

**Affiliations:** ^1^Department of Pharmacy, Nanjing Drum Tower Hospital, The Affiliated Hospital of Nanjing University Medical School, Nanjing, China; ^2^Department of Stomatology, Children's Hospital of Nanjing Medical University, Nanjing, China; ^3^School of Pharmacy, China Pharmaceutical University, Nanjing, China; ^4^Department of Pharmacy, The Cancer Hospital of the University of Chinese Academy of Sciences (Zhejiang Cancer Hospital), Institute of Cancer and Basic Medicine (IBMC), Chinese Academy of Sciences, Hangzhou, China

**Keywords:** STING, cGAS, inflammation, cardiovascular diseases, therapy

## Abstract

Sterile inflammation characterized by unresolved chronic inflammation is well established to promote the progression of multiple autoimmune diseases, metabolic disorders, neurodegenerative diseases, and cardiovascular diseases, collectively termed as sterile inflammatory diseases. In recent years, substantial evidence has revealed that the inflammatory response is closely related to cardiovascular diseases. Cyclic guanosine monophosphate–adenosine monophosphate synthase (cGAS)-stimulator of interferon genes (STING) pathway which is activated by cytoplasmic DNA promotes the activation of interferon regulatory factor 3 (IRF3) or nuclear factor-κB (NF-κB), thus leading to upregulation of the levels of inflammatory factors and interferons (IFNs). Therefore, studying the role of inflammation caused by cGAS-STING pathway in cardiovascular diseases could provide a new therapeutic target for cardiovascular diseases. This review focuses on that cGAS-STING-mediated inflammatory response in the progression of cardiovascular diseases and the prospects of cGAS or STING inhibitors for treatment of cardiovascular diseases.

## Introduction

The first line of defense in mammals is orchestrated by the innate immune system which recognizes various pathogens and damage-associated molecular patterns (PAMPs and DAMPs) through pattern recognition receptors (PRRs) (1). DNA, RNA, lipopolysaccharide (LPS), peptidoglycan, and other components produced by viruses, bacteria, and other invading microorganisms comprise PAMPs, whereas high mobility group box 1 (HMGB1), endogenous DNA, and other substances induced by cellular stress are called DAMPs (2). DNA receptors such as cGAS which acts as an important PRR in the cytoplasm and recognizes free cytoplasmic DNA activate STING by synthesizing 2′-3′-cyclic GMP-AMP (2′-3′-cGAMP), indicating that cGAS-STING pathway plays a critical role in the innate immune response (1, 3–5). Activated STING is transported by vesicles through the endoplasmic reticulum-Golgi intermediate compartment (ERGIC) and activates TANK binding kinase 1 (TBK1), interferon regulatory factor 3 (IRF3), nuclear factors-κB (NF-κB), and other downstream signaling molecules (6–9). Activation of IRF3 and NF-κB can upregulate the levels of IFNs, interferon stimulated genes (ISGs), and inflammatory factors, however, in a large number of conditions, excessive inflammation causes damage to host tissue and leading to organ dysfunction, which in turn regulate the progression of multiple autoimmune diseases, metabolic disorders, neurodegenerative diseases, and cardiovascular diseases, collectively termed as sterile inflammatory diseases (10–13). Inflammatory processes crucially regulate the onset, progression and outcomes of cardiovascular diseases (13). In the onset and progression of atherosclerosis, a large number of mediators which regulate the inflammatory processes play pivotal roles (14). Coincidentally, in the pathogenesis of heart failure, the persistent inflammatory response is functionally important for contributing to adverse outcomes (15). Yearly, the important role of cGAS-STING signaling pathway in sterile inflammation has historically been appreciated (11). Emerging evidence supports that targeting cGAS-STING-mediated inflammation can be effective in treatment for cardiovascular diseases (16–21). This review focuses on the role of cGAS-STING-mediated sterile inflammation in cardiovascular diseases as well as the discovery of cGAS and STING inhibitors.

## The activation of DNA-cGAS-STING-mediated inflammation in cardiovascular diseases

Under cardiovascular pathological conditions, disrupting DNA compartmentalization and/or its metabolism leads to cGAS activation (22, 23). Cytoplasmic DNA is accumulated by a variety of ways, including the efflux of mtDNA and nuclear DNA (micronuclei) into cytosol induced by membrane integrity following mitochondrial stress and chromosomal damage, extracellular DNA released from dying cells, DNA aggregation caused by loss-of-function gene mutations in the nucleases (DNase I, DNase II, TREX1, and RNase H2) (24–26). ELISA, LC-MS, and LC-MS/MS are the main approaches to detect the content of cGAMP in heart or vascular tissues (16, 21, 27, 28). Immunofluorescene staining with dsDNAand qPCR of cytosolic mtDNA are the main approaches to detect the content of cytosolic DNA in heart or vascular tissues (21, 28–35). Oxidative stress, mitochondrial damage, and mtDNA leakage are considered to be the main reason for generation of cGAMP or accumulation of cytosolic DNA in cardiovascular diseases (16, 21, 29–35). Herein, we summarize the studies that have uncovered the increased content of cGAMP or cytoplasmic DNA in cardiovascular diseases (Table 1).

**Table 1:** cGAMP or cytosolic DNA was detected in experimental models of cardiovascular diseases.

	**Elevated indicator**	**Test sample**	**How to detect**	**The reason for the elevated level of cGAMP or cytosolic DNA**	**Diseases**	**Changes in cGAS-STING pathway**	**Experimental model**	**Reference**
Heart disease	cGAMP	Heart tissue	ELISA assays	Oxidative stress	Diabetic cardiomyopathy	Increased expression of cGAS, elevated phosphorylation of TBK1 and IRF3	STZ and HFD-induced mice	(16)
	cGAMP	Heart tissue	LC-MS	Impaired mitophagy	Inflammation	Elevated levels of ISGs and inflammatory cytokines	exhaustive exercise-induced Prkn^−/−^ Pink^−/−^ mice	(27)
	Cytosolic mtDNA	Heart tissue	qPCR quantified mtDNA release	Mitochondrial damage	Smoke exposure-induced cardiac anomalies	Increased expression of cGAS and STING	Mice following side-stream smoke exposure	(30)
	Cytosolic DNA	Heart tissue	Immunofluorescence double staining with anti-dsDNA and motifilin	mtDNA leakage	Diabetic cardiomyopathy	Increased expression of cGAS and STING, elevated phosphorylation of TBK1 and IRF3	HFD-fed db/db mice	(34)
	Cytosolic DNA	Cardiomyocyte	Immunofluorescence double staining with anti-dsDNA and motifilin	mtDNA leakage	Diabetic cardiomyopathy	Increased expression of cGAS and STING, elevated phosphorylation of TBK1 and IRF3	PA-stimulated H9C2 cells	(34)
Vascular disease	cGAMP	Atherosclerotic lesion	LC-MS/MS	DNA damage	Atherosclerosis	Increased expression of STING	Atherosclerotic patients, western-type diet-induce Apoe^−/−^ mice	(21)
	cGAMP	Endothelial cells	LC-MS	Transfection with DNA	Endothelial dysfunction	Elevated phosphorylation of STING, TBK1, and IRF3	Plasmid DNA or mtDNA-stimulated hLMVECs	(28)
	Cytosolic DNA	Aortic tissue	Immunofluorescence double staining with anti-dsDNA and anti-Tommo20	Oxidative stress	Aortic aneurysm and dissection	Elevated expression and phosphorylation of STING, TBK1, and IRF3	Patients with ascending thoracic aortic and dissection, HFD and angiotensin II-challenged mice	(29)
	Cytosolic DNA	Vascular smooth muscle cells	Immunofluorescence double staining with anti-dsDNA and anti-Tommo20	Oxidative stress	Aortic aneurysm and dissection	Elevated expression and phosphorylation of STING, TBK1, and IRF3	H_2_O_2_-stimulated aortic SMCs	(29)
	Cytosolic DNA	Endothelial cells	Immunofluorescence double staining with anti-dsDNA and mitoTracker	Mitochondrial damage	Endothelial inflammation	Elevated phosphorylation of IRF3	PA-stimulated human aortic ECs	(35)
	Cytosolic DNA	Atherosclerotic lesion	Immunogold staining with dsDNA	DNA damage	Atherosclerosis	Increased expression of STING	western-type diet-induce Apoe^−/−^ mice	(21)
	Cytosolic mtDNA	Endothelial cells	qPCR of mtDNA in cytosolic fraction	Mitochondrial damage	Impaired angiogenesis	Increased expression of cGAS and STING, elevated phosphorylation of IRF3	PA-stimulated human aortic ECs	(31)
	Cytosolic mtDNA	Endothelial cells	qPCR of cytosolic mtDNA	GSDMD-mediated mtDNA leakage	Endothelial dysfunction	Not detected	LPS-stimulated hLMVECs	(28)
	Cytosolic mtDNA	Endothelial cells	qPCR of cytosolic mtDNA	Mitochondrial damage	Endothelial-to-mesenchymal transition	Increased expression of cGAS and STING, elevated phosphorylation of IRF3	PA-stimulated human aortic ECs	(32)
	Cytosolic DNA	Macrophage	ELISA assays	Not given	Atherosclerosis	Not detected	oxLDL-stimulated J774.A1 cells	(33)

Recognizing cytoplasmic DNA by cGAS, generated cGAMP binds to STING and induces the formation of STING dimer (4). Thus, reticulum STING which promotes the recruitment and activation of TBK1 is transported to the Golgi apparatus *via* the ERGIC, where palmitoylation of its Cys88 and Cys91 sites further promotes the recruitment and activation of TBK1, thereby activating IRF3 and NF-κB (36, 37). Nuclear transcription of IRF3 or NF-κB promotes the expression of downstream inflammatory factors such as TNF-α, IL-6, IL-1β, MCP-1, and IFNs, eventually leading to the inflammatory response, suggesting activation of IRF3 or NF-κB play an important role in sterile inflammatory diseases (9, 12, 22, 24, 38–40). Furthermore, the C- terminal tail (CTT) is necessary for STING to activate TBK1 and IRF3, and there is a conservative consensus motif in the CTT (pLxIS; p is hydrophilic and x is any residue), which is phosphorylated at Ser366 in human STING (Ser365 in mice) (41, 42). This phosphorylation is mediated by TBK1, which activates IRF3. In addition, STING which is phosphorylated at Ser374 in human STING (Ser373 in mice) activates inhibitor of nuclear factor-κB kinase (IKK) during endoplasmic reticulum translocation, resulting in the phosphorylation of inhibitor of nuclear factor-κB (IκB) through ubiquitin-proteasome degradation and the release of free NF-κB (43–45). Additionally, E3 ubiquitin ligase TNF receptor associated factor 6 (TRAF6) mediates the linkage of STING to K63 multiubiquitin chains, which then activates NF-κB *via* the TGF-β activated kinase-1(TAK1)/TAK1 binding protein2/3(TAB2/3)/IKK pathway (46). These data suggested that STING might directly activate NF-κB to induce the inflammatory response. However, contrary to this, it has been reported that STING activates NF-κB through TBK1 (47, 48). At present, this is a controversial viewpoint that needs further exploration.

**Figure 1:**
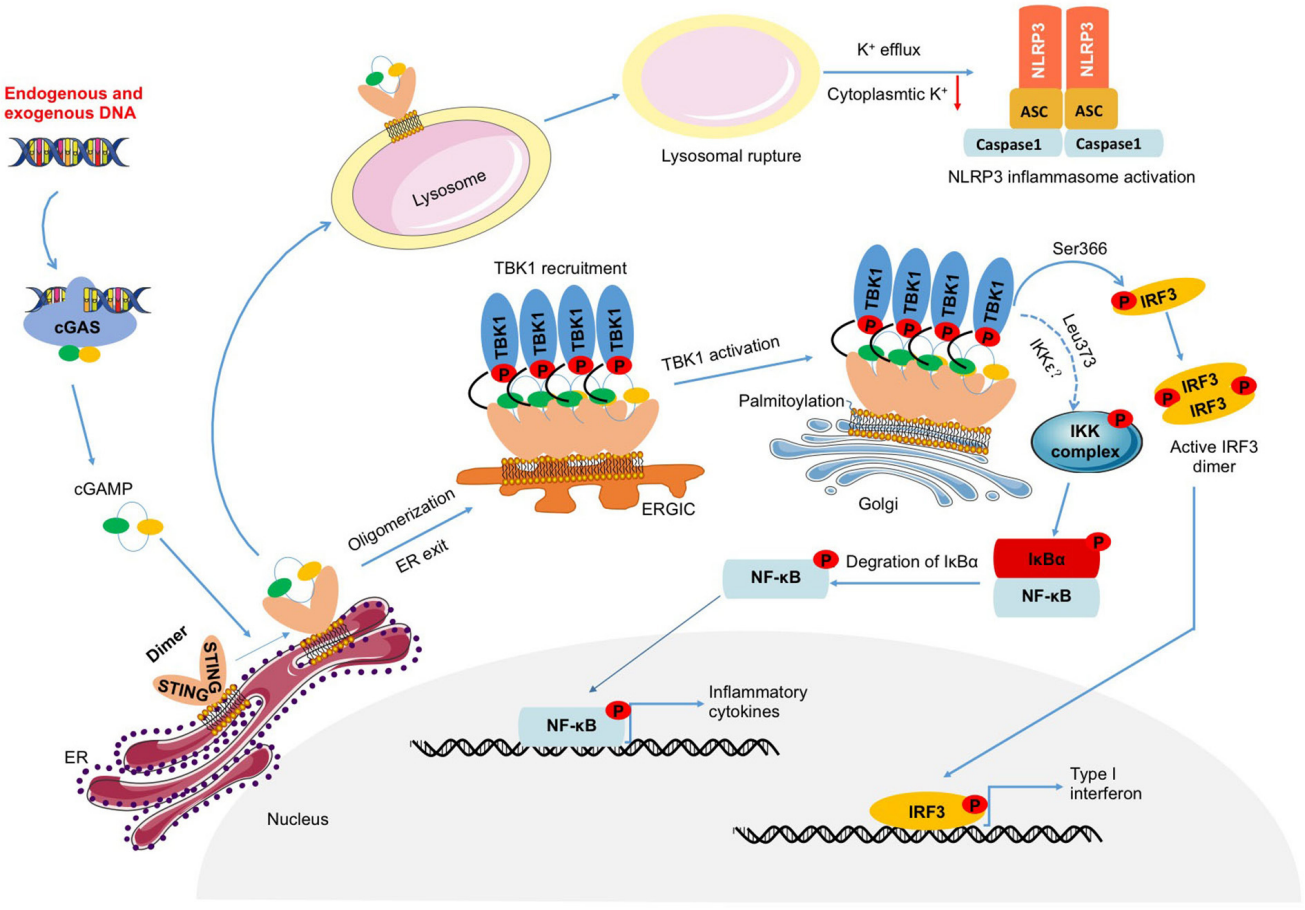
DNA-cGAS-STING-mediated inflammation. Endogenous or exogenous DNA leads to cGAS activation, which catalyzes the production of cGAMP. Activated STING induced by cGAMP not only triggers the activation of TBK1-IRF3 and NF-κB which induce the transcription of IFNs and inflammatory factors, but also prompts NLRP3 inflammasome activation through lysosomal rupture-induced K^+^ efflux.

In summary, STING is an important intracellular adaptor protein that mediates the cellular inflammatory immune response (Figure 1), and exploring the STING-mediated inflammatory response will help to uncover the role of STING in inflammatory diseases.

### IRF3-mediated inflammation

Cryo-electron structural data imply that TBK1 is able to phosphorylate the CTT of STING of an adjacent STING dimer, rather than the CTT of its own dimer (41). During ER exit of STING, STING oligomer is transported by vesicles through the endoplasmic reticulum-Golgi intermediate compartment (ERGIC), where brought into close proximity to the catalytically active IRF3 (8, 49). Phosphorylated IRF3 forms a dimer that enters the nucleus and binds to specific gene promoters to promote up-regulation of IFNs, ISGs, and inflammatory factors. IRF3 was reported to be involved in the pathological process after MI, whereas there was a decrease in cardiomyocyte apoptosis in the IRF3-deficient mouse model, which further improved the remodeling after MI (50). Additional studies showed phosphorylated IRF3 subsequently translocated into nucleus and increased the expression of NOD-like receptor protein 3 (NLRP3), leading to the development of sepsis and sepsis-induced cardiomyopathy (19). Furthermore, adioprotective 105 kDa protein (RP105), a negative regulator of TLR4, which inhibited transcriptional activity of IRF3, performed a protective role in myocardial ischemia reperfusion injury by anti-apoptosis approach (51). These evidences indicated IRF3 was involved in the pathological cardiomyopathy.

Chronic activation of STING-IRF3-mediated inflammation contributes to inflammatory cardiovascular diseases. In fact, free fatty acids cause activation of the STING-IRF3 pathway and an increase in adhesion factors such as vascular cell adhesion molecule 1 (VCAM-1) and intercellular adhesion molecule 1 (ICAM-1) in endothelial cells, which can be reversed by STING knockout (35). Moreover, a moderate-to-strong immunoreactivity effect associated with IRF3 in the endothelium and macrophages of the atherosclerotic plaques in patients with coronary heart disease and in hyperlipidemic mice (52). Nevertheless, IRF3 deficiency suppresses the secretion of VCAM-1 and the expression of ICAM-1, which subsequently attenuates macrophage infiltration in HFD-induced Apoe^−/−^ mice (52). The above inflammatory factors mediated by the STING-IRF3 pathway induce the inflammatory response, resulting in different degrees of injury to organs.

### NF-κB-mediated inflammation

STING activates IKK complex on the Golgi apparatus and phosphorylates the transcription factor IκB, resulting in its degradation through the ubiquitin-proteasome pathway, releasing free NF-κB into the nucleus (43, 48, 53). In keratinocyte, DNA damage signals are transmitted to TRAF6 to activate STING. In this process, TRAF6 acts as an E3 ubiquitin ligase to mediate the linkage of STING to K63 multiubiquitin chains. K63 multiubiquitin chains assemble TGF-β activated kinase-1 (TAK1), TAK1 binding protein2/3 (TAB2/3), and IKK to activate NF-κB and up-regulate inflammation (46). Although many studies have shown that STING directly activates NF-κB and causes inflammation, there is also literature indicating that STING activates NF-κB through the TRAF6-TBK1 axis. Two studies from different research groups showed that IKKε as an isoenzyme of TBK1 involved in STING-mediated NF-κB activation (43, 45). Therefore, whether STING-NF-κB is mediated by TBK1 remains to be further clarified. One thing is certain that phosphorylation of STING at Leu373 is critical for NF-κB activation (44, 45), however, the signal transduction pathway remains to be seen in the future.

In high-fat diet-induced mice, the STING-NF-κB pathway is activated in kupffer cells and inflammatory factors such as IL-6, IL-1β, and TNFα are increased. However, the inflammatory response in the livers of mice is reversed by STING knockout or NF-κB inhibitor (54, 55). Recently, activation of the STING-NF-κB pathway has been found in mouse models of acute and chronic kidney injury (56, 57). In the acute kidney injury of cisplatin-induced mice, mitochondrial damage in renal tubular epithelial cells leads to the leakage of mitochondrial DNA into the cytoplasm and activation of the cGAS-STING-NF-κB pathway, eventually contributing to the upregulation of inflammatory factors (57). Consistently, renal tubular cell-specific transcription factor A (TFAM) knockout activates the STING-NF-κB pathway, leading to chronic kidney inflammation (57). In summary, the STING-NF-κB pathway activates classic inflammatory factors such as IL-6 and TNF-α and induces the inflammatory response.

### NLRP3-mediated inflammasome activation

NLRP3 inflammasome activation which promotes pro-inflammatory cytokines secretion and cysteinyl aspartate specific proteinase (Caspase) activation plays a vital role in the innate immune system (58, 59). Moreover, in sensing DAMPs, increased nuclear localization of pro-Caspase-1 and activated Caspase 1 upregulated inflammatory genes in lysophosphatidylcholine-indued human aortic endothelial cells (HAECs) (60). Moritz et al. reported cGAS/STING signaling activates NLRP3 inflammasome independently of typeIinterferom (61). Mechanistically, STING trafficking leads to lysosomal membrane permeabilization and a lytic form of lysosomal cell death, thereby inducing the efflux of K^+^. This subsequently leads to a decline in cytosolic K^+^, thereby triggering the activation of NLRP3/apoptosis associated speck-like protein (ASC)/Caspase-1 inflammasome, which promotes sterile inflammation *via* mediating the maturation and release of IL-1β and IL-18 (61). In fact, in LPS-induced mice, genetic deletion of STING reduced the expression of NLRP3 and activation of NLRP3/ASC/Caspase-1inflammasome, which reduced myocardial inflammation (19). Subsequent *in vitro* experiments revealed that the protective effects of STING knockdown in LPS-induced cardiomyocytes were reversed by NLRP3 overexpression (19). Additionally, the cGAS-STING signaling pathway was activated in diabetic hearts, which leads to the activation of the NLRP3 inflammasome and proinflammatory cytokine release. However, STING knockdown *via* adeno-associated virus-9 (AAV9) in diabetic mouse heart alleviated cardiac pyroptosis and the inflammatory response, thereby attenuating the progression of diabetic cardiomyopathy (16). Moreover, analysis of differentially expressed genes showed that NLRP3 inflammasome-related genes including *Nlrp3, Gsdmd, Caspase1, Il1b, Il18* were reduced by genetic deletion of STING in RNA-sequencing (RNA-seq) analysis performed in ascending aortas from wild-type mice and *Sting*^gt/gt^ mice that were unchallenged or challenged with HFD and angiotensin II (Ang II) infusion (29). Notably, it was observed that, NLRP3 inflammasome activation dependent on cGAS-STING signaling fueled myocardial inflammation and the development of cardiovascular diseases.

## cGAS-STING-mediated inflammation promotes the pathological process of cardiovascular diseases

**Figure 2:**
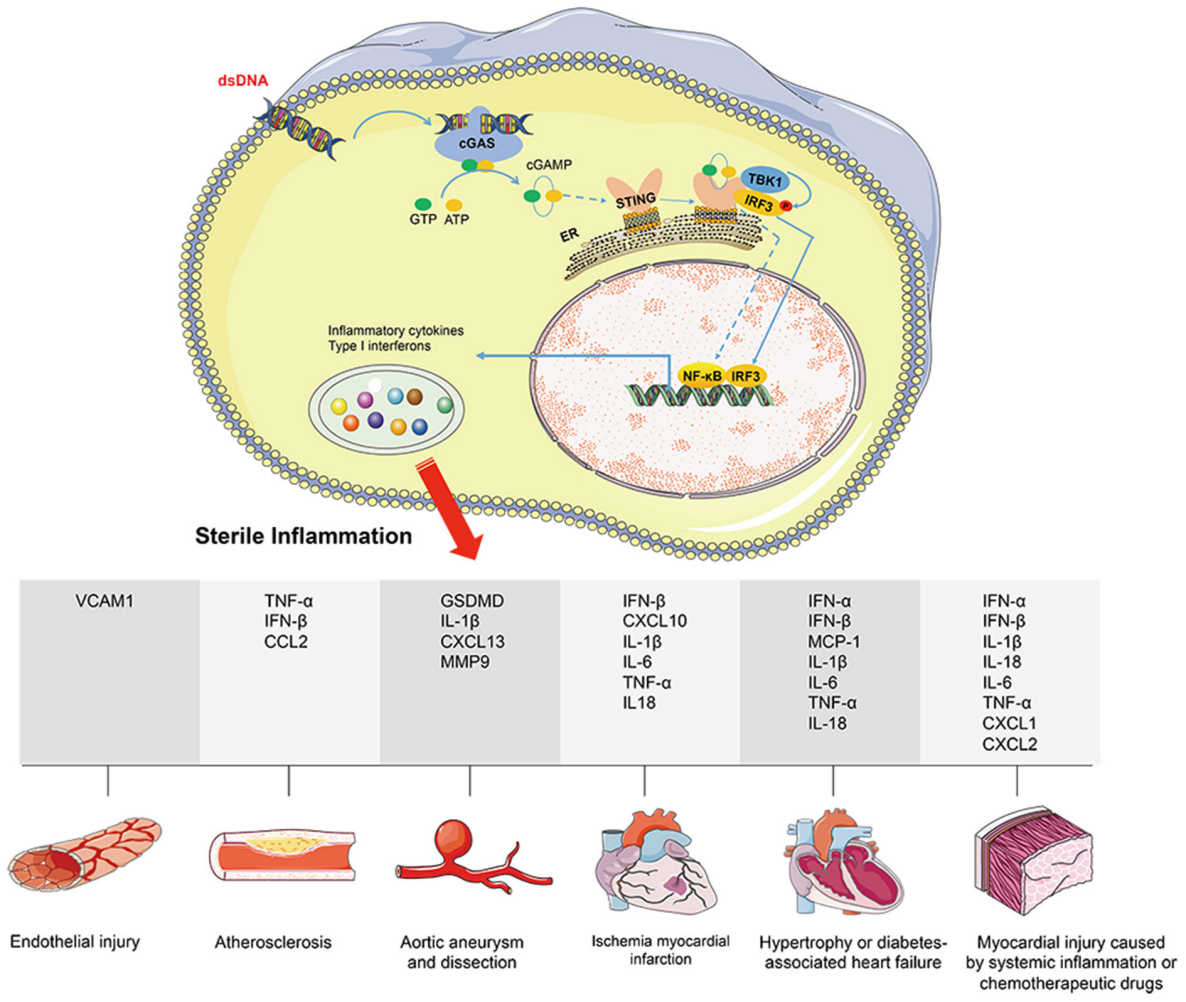
cGAS-STING signaling activation mediates cardiovascular diseases *via* promoting sterile inflammation. Under cardiovascular pathological conditions, disrupting DNA compartmentalization and/or its metabolism leads to cGAS-STING signaling activation. Nuclear transcription of IRF3 or NF-κB promotes the expression of downstream inflammatory factors (including VCAM1, GSDMD, CCL2, CXCL1, CXCL2, CXCL10, CXCL13, TNF-α, IL-1β, IL-6, IL-18, MCP-1, MMP9, IFN-α, and IFN-β), eventually leading to the inflammatory response. Herein, the pathological process of cardiovascular diseases regulated by cGAS-STING-driven inflammation includes endothelial injury, atherosclerosis, aortic aneurysm and dissection, ischemia myocardial infraction, hypertrophy or diabetes associated heart failure, and myocardial injury caused by systemic inflammation or chemo therapeutic drugs.

Inflammation is closely related to the occurrence of cardiovascular diseases. In recent years, uncovering the STING-mediated inflammatory response has advanced the study of cardiovascular diseases. The approaches to cytoplasmic DNA accumulation and how to activate cGAS-STING-mediated inflammatory response under cardiovascular pathological conditions have been summarized respectively. This section mainly reviews the pathological process of cardiovascular diseases regulated by cGAS-STING-driven inflammation (Figure 2).

### Vascular injury

It has been reported that patients with STING-associated vasculopathy with onset in infancy (SAVI) were characterized by systemic inflammation, severe cutaneous vasculopathy, and interstitial lung disease, which caused by gain-of-function mutations in Tmem173 (also called Sting1), including V147L, N153S, V155M, and V155R (62). These mutations promote the aggregation and activation of STING from the endoplasmic reticulum to perinuclear vesicles without ligands, thereby activating the STING-IRF3 pathway and increasing the expression level of type | IFNs, which in turn promotes inflammation (63, 64). Consistently, STING N153S or V154M knock-in mouse model demonstrates that SAVI-associated STING mutations cause inflammatory lung and skin disease (63, 64). However, STING N153S in mice causes a systemic inflammatory response independent of IRF3 by impacting T cells at the early stages of thymocyte development (63).

A change in vascular permeability is the critical progression of the lethal process of sepsis. LPS causes pyroptosis of vascular endothelial cells, and the increased activation of gasdermin D (GSDMD) promotes the release of mitochondrial DNA, thereby activating the cGAS-STING pathway, inhibiting endothelial cell proliferation, and ultimately leading to a change in vascular endothelial permeability (28). Nonetheless, exogenous mitochondria exposure induces endothelial STING activation, promoting effector memory T cell adhere to endothelial cell, which was reversed by STING inhibitor (65). Mechanically, different from canonical cGAS signaling, mitochondria-induced endothelial STING activation which was mediated by IFN-inducible factor 16 (IFI16) triggered the increases of NF-κB-mediated adhesion molecules (65). Besides, as an important driver of vascular inflammation, endothelial cells play an important role in the onset of vascular injury or chronic metabolic disease-associated tissue inflammatory injury (66). Obesity is a metabolic disorder that fosters the occurrence and complication of diverse disease, which goes along with inflammation (67, 68). Increasing evidence showing that increased plasma free fatty acids levels induced by obesity and type 2 diabetes play detrimental roles in the pathogenesis of cardiovascular diseases (69). Obesity leads to an increase in the level of free fatty acids in the peripheral circulation, which promotes the leakage of mitochondrial DNA from vascular endothelial cells into the cytoplasm, which activates the cGAS-STING-IRF3 pathway and up-regulates the expression of ICAM-1, eventually contributing to vascular endothelial inflammation (35). These evidences presented above demonstrate that STING-mediated inflammation is involved in endothelial injury.

### Atherosclerosis

Atherosclerosis (AS) is a chronic inflammatory disease, which is the main cause of clinical cardiovascular events. It's reported that atherosclerotic plaque macrophage-derived oxidized mitochondrial DNA induced STING-dependent inflammation, eventually leading to exacerbation of atherosclerosis (21). Pham PT et al. also found the accumulated content of cytoplasmic DNA and the elevated levels of cGAMP caused by DNA damage in atherosclerotic plaque of HFD-fed *Apoe*^−/−^ mice (21). Further studies revealed that activation of cGAS-STING signaling cytoplasmic sensing in macrophage triggered persistent vascular inflammation and induction of multiple inflammatory factors (21). Mechanically, this process might be mediated by transactive response DNA-binding protein-43 kDa (TDP43)-induced mitochondrial DNA release (70). Nonetheless, genetic deletion of STING macrophage-derived or pharmacological blockade of STING reduced inflammatory molecule expression and macrophage infiltration, thereby resulting in the alleviation of the progression of atherosclerosis in *Apoe*^−/−^ mice fed with HFD (21, 71).

In addition, atherosclerotic plaques consist of a surface fibrous cap and an inner lipid core composed of abundant lipids and necrotic cells, where the development of VSMC phenotypic transformation play a vital role (72). Chronic kidney diseases (CKD) promote premature aging of VSMCs and cause it to undergo a phenotype transformation as a result of autocrine/paracrine activation, resulting in the loss of vascular smooth muscle cells in the fibrous cap and a thinning of the fibrous cap, which accelerates atherosclerotic plaque rupture (73). Furthermore, CKD-induced oxidative stress leads to mitochondrial damage and mitochondrial permeability transition pore (mPTP) opening in VSMCs, resulting in the release of mitochondrial DNA into the cytoplasm and triggering the inflammatory response through the cGAS-STING pathway (74). The above studies have shown that intracellular DNA accumulation promotes the development of atherosclerosis by activating the STING-mediated inflammatory response.

### Aortic aneurysm and dissection

Aortic aneurysms and dissections (AAD) are preceded by ECM rupture and a progressive loss of VSMCs, which eventually results in AAD form and the aorta rupture. Wei L et al. found that dsDNA from aortic VSMCs leaked into the cytoplasm to activate STING in human and mouse AAD, resulting in necrosis of VSMC (29). Moreover, dsDNA releases into the vascular wall to recruit macrophages and activates the STING-TBK1-IRF3 pathway in macrophage (29). Then, the expression of matrix metalloproteinase-9 (MMP9) is upregulated through IRF3 directly binding to the promoter of MMP9, which leads to damage the vascular elastic plate and promote the AAD process (29).

### Myocardial infarction and hypertrophy-associated heart failure

Pressure overload and ischemia are pivotal pathophysiological causes of heart failure and myocardial infarction (MI) (75). Accumulated experiments have proven that overactive inflammation induced by MI contributed to the increased size of cardiomyocyte and myocardial remodeling, resulting in left ventricular systolic dysfunction (76). In fact, response to MI, ischemic cell death and uptake of exogenous DNA by macrophage fuel an acute inflammation, eventually resulting in left ventricular dysfunction and death (20, 50). Mechanically, Cao et al. found that the leakage of nucleic acids to cytoplasmic induced by ischemic myocardial injury activated the cGAS-STING signaling pathway, resulting in M1-like polarization of macrophages and the induction of inflammatory programs with increased levels of NLRP3, Caspase1, IL-1β, IL-6, IL18, TNF-α, whereas inhibition of STING or cGAS promotes the M2 transformation of recruited macrophages toward repair, which is crucial to the recovery of MI (20). On another hand, single-cell RNA-seq analysis from myocardial tissues of MI mice showed that cardiac resident macrophage-derived IRF3-IFN axis provoked the expression of inflammatory cytokines and chemokines (including TNF-α, IL-1β, IL-6, IFN-β, and CXCL-10) and inflammatory cell infiltration into myocardium, whereas interruption of IRF3 signaling by IFNAR-neutralizing antibody or genetic deletion of cGAS, STING, or IRF3 reversed these changes (50). Similarly, myocardial ischemia-reperfusion led to cardiomyocytes release DNA and HMGB1, which enter the circulation to activate the inflammatory response (77). Correspondingly, blocking the macrophage-derived type I IFNs signaling pathway by IFN antibody, STING antibody or cGAS inhibitor in myocardial ischemia-reperfusion mice can markedly reduce infarct size (77).

Heart failure is an end-stage clinical syndrome of cardiovascular disease, which is characterized by cardiac systolic or diastolic dysfunction and impaired ejection. Elevated levels of STING, IFNα, and IFNβ have been found in human samples of dilated and hypertrophic cardiomyopathy (78). In the hearts of transverse aortic constriction (TAC) mice, there is increased expression of STING, IFNα, and IFNβ, however, STING knockout markedly improves cardiac function in these mice (78). Moreover, 3 days after TAC surgery, the expression levels of IFNs, CXCL10, IFIT3, and ISG15 in the mouse myocardium are significantly increased, and further knockdown of cGAS using adeno-associated virus (AAV9) considerably reduces left ventricular remodeling and fibrosis in these mice (79). These studies demonstrate that released DAMPs promote the STING-mediated inflammatory response and the pathological process of heart failure and myocardial infarction.

### Diabetic cardiomyopathy

Diabetes as a chronic disease, long-term accumulation of cardiac pressure overload may lead to heart failure (80). Recently, STING has been reported to be involved in islet damage, cholesterol metabolism and liver inflammation (39, 54, 55, 70, 81–83), thus it can be seen that cGAS-STING signaling is closely related to diabetic cardiomyopathy. Diabetic cardiomyopathy has occurred from time to time in clinical practice, and inflammation plays a crucial role in its development. Yan et al. reported that NLRP3 inflammasome-induced pyroptosis caused by the activation of cGAS-STING signaling was participated in the development of diabetic cardiomyopathy (16). Hyperlipidemia in diabetic mice caused DNA leakage of myocardial cells to activate cGAS-STING signaling, which led to pyroptosis and induced inflammation, ultimately resulting in myocardial hypertrophy and remodeling (16). Nonetheless, knockdown of *Sting* gene by AAV9 or pharmacological inhibition of STING effectively alleviated myocardial inflammation and diabetic cardiomyopathy (16, 34). In conclusion, it elucidated the critical role of cytosolic mtDNA-induced cGAS-STING activation in the pathogenesis of obesity-related DCM and provided preclinical validation as a new potential therapeutic strategy for the treatment of DCM.

### Myocardial injury caused by systemic inflammation or chemotherapeutic drugs

STING is involved in mediating systemic inflammation caused by risk factors that lead to myocardial damage, such as smoking, systemic lupus erythematosus (SLE), and sepsis (4, 30, 84, 85). It has been reported that side-flow smoke causes mitochondrial damage in cardiomyocytes, which triggers the release of mitochondrial DNA into the cytoplasm, leading to activation of the STING pathway and the development of an abnormal cardiac structure and cardiac dysfunction (30). Herein, knockout of Beclin 1, which is involved in autophagosome formation and mitochondrial DNA clearance, exacerbates the STING-mediated inflammatory response and cardiac dysfunction induced by side-flow smoke (30). Under physiological conditions, DNase III/three prime repair exonuclease 1 (TREX1) can remove cytoplasmic DNA and prevent endogenous DNA accumulation (84). Inactivating mutations in TREX1 might lead to SLE. Since it is an autoimmune disease, SLE patients are more prone to cardiovascular diseases than healthy people, and one third of SLE deaths are caused by cardiovascular events (85–87). Deficiency of TREX1 induced high levels of IFNs through activation of the STING-IRF3 pathway, leading to myocardium, vasculitis, and other diseases (85, 88). cGAS knockout can inhibit the above inflammatory reactions (4). On the other hand, LPS-induced septic cardiomyopathy has been shown to have the characteristic with cardiac dysfunction and inflammation (89–91). Nevertheless, deficiency of STING considerably was found to improve cardiac function and inflammation in mice (19). Furthermore, deficiency of STING suppressed NLRP3/Caspase1-mediated pyroptosis induced by LPS, thereby inhibiting the generation of mature IL-1β and IL-18 (19). The above studies have confirmed that STING mediates the induction of myocardial tissue inflammation and causes myocardial injury in systemic inflammation.

In many clinical patients treated with chemotherapy drugs, long-term chemotherapy has been observed to promote cardiac insufficiency years later (92–95). Cisplatin is a broad-spectrum chemotherapy drug that has been clinically found to cause myocardial damage (92, 94). Our previous study found that the expression of inflammatory factors, such as TNF-α and IL-6, is upregulated in the myocardial tissues of cisplatin-induced mice (96). However, genetic deletion of STING could effectively inhibit the expression of myocardial inflammatory factors and cardiac dysfunction induced by cisplatin (96). Thus, the role of the STING-mediated inflammatory response in myocardial injury induced by chemotherapy drugs deserves more attention.

## Discovery of drugs targeting cGAS-STING

As mentioned above, cGAS-STING signaling participated in the development of multiple sterile cardiovascular diseases. Therefore, targeted inhibition of cGAS or STING provides new avenues for the treatment of cardiovascular diseases. Here, this section mainly discusses the cGAS or STING inhibitors applied in cardiovascular diseases (Table 2).

**Table 2:** STING or cGAS inhibitors proven to exert protective effects in experimental models of cardiovascular diseases.

	**Compound**	**Structure**	**Target**	**Molecular mechanism**	**Cardiovascular disease**	**Experimental model**	**Reference**
Heart disease	Astin C	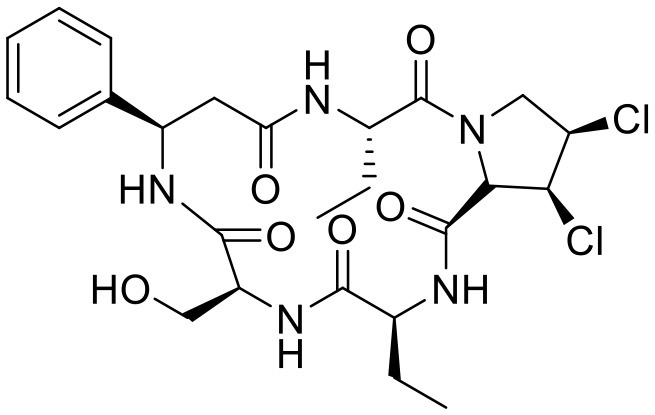	STING	Targeting the CDN-binding domain	Cardiac anomaly	PA-induced cardiomyocyte	(113)
	Nitro-fatty acids	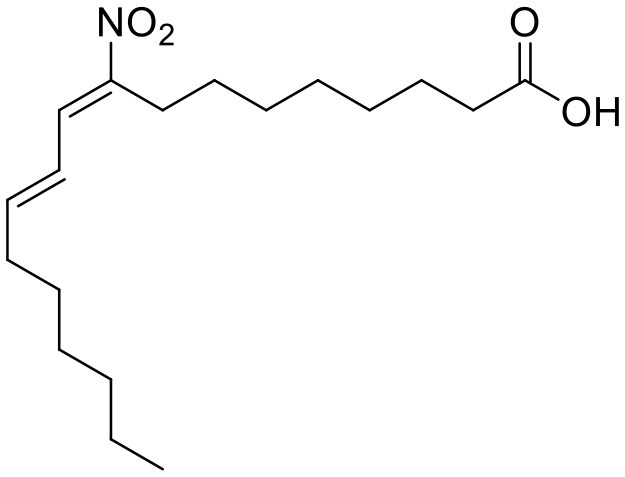	STING	Targeting the palmitoylation site	Ischemic ventricular arrhythmias; cardiac remodeling	Left anterior descending artery (LAD) ligation; angiotensin II infusion;	(119, 120)
	C-176	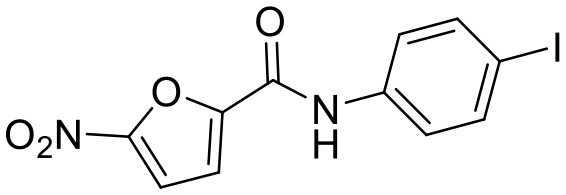	STING	Targeting the palmitoylation site at Cys91	Diabetic cardiomyopathy	HFD-fed db/db mice	(34)
	H-151	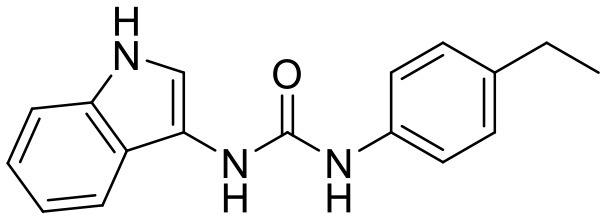	STING	Targeting the palmitoylation site at Cys91	Myocardial infarction; myocardial ischemia-reperfusion injury	LAD; myocardial ischemia-reperfusion	(17, 18)
	RU.521	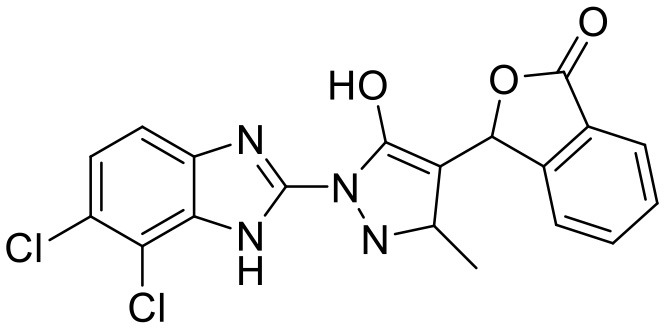	cGAS	Targeting the catalytic site	Septic cardiomyopathy	LPS-induced sepsis	(112)
	PF-06928215	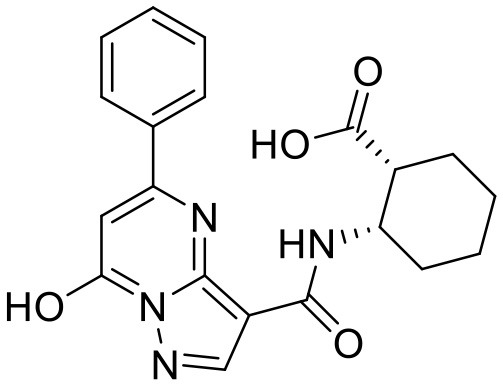	cGAS	Targeting the catalytic site	Cardiac anomaly	PA-induced cardiomyocyte	(113)
Vascular disease	Nitro-fatty acids	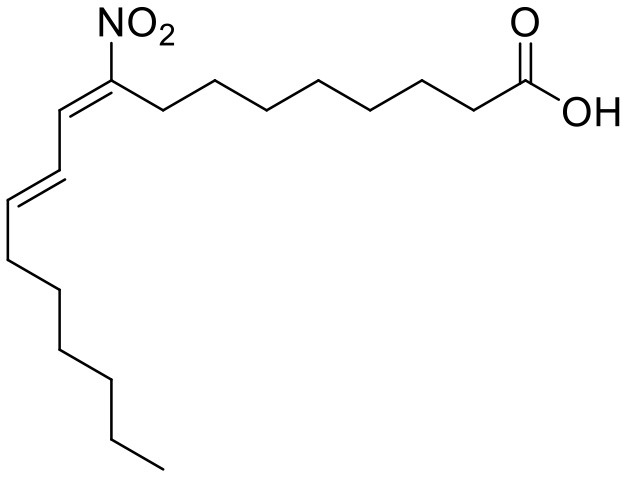	STING	Targeting the palmitoylation site	Abdominal aortic aneurysm; pulmonary hypertension; neointima formation; endothelial injury	PCSK9-D377Y induced hypercholesterolemia with angiotensin II infusion; high-fat diet (HFD) or hypoxia-induced mice; wire-mediated vascular Injury; Inflammatory factors-induced MS-1 cells	(121–124)
	C-176	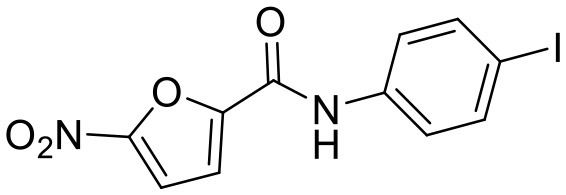	STING	Targeting the palmitoylation site at Cys91	Atherosclerosis	Western-type diet-induce Apoe–/- mice; CKD-induced Apoe^−/−^ mice fed with HFD	(21, 74)
	Suramin	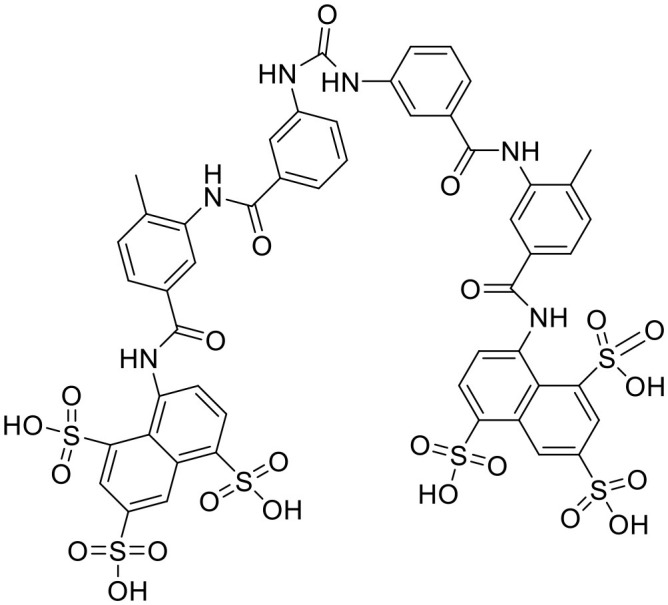	cGAS	Inhibiting the binding of DNA to cGAS	Neointima formation; pulmonary hypertension	Vessel grafting-induced carotid artery; FAM3A or PDGF-AB-induced VSMCs; Monocrotaline-induced rats	(105–108)
	A151	5'-TTAGGGTTAGGGTTAGGGTTAGGG-3'	cGAS	Inhibiting the binding of DNA to cGAS	Atherosclerosis	Normal diet-induced Apoe^−/−^ mice	(104)
	Perillaldehyde	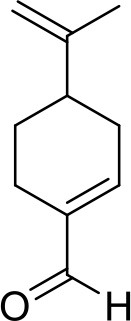	cGAS	Inhibiting the binding of DNA to cGAS	Angiogenesis; atherosclerosis	Sunitinib-injured zebra-fish embryos; HFD-induced rat and Apoe^−/−^ mice	(109, 110)

The development strategy of small molecule inhibitors targeting cGAS is mainly through the following three mechanisms: (1) mediate post-translational modification (PTM) of cGAS (97); (2) block the binding of DNA to cGAS (98); (3) occupy the cGAS catalytic pocket (99). Aspirin, targeting the acetylation of cGAS at Lys 384, Lys394, and Lys414, effectively suppressing autoimmunity induced by genetic deletion of *Trex1* (100). Inhibitors that target the binding of DNA to cGAS include hydroxychloroquine (HCQ), quinacrine (QC) and X6, which belong to the first discovered inhibitor of cGAS (101, 102). Additionally, synthetic oligonucleotides (ODNs) competitively inhibit the binding of DNA to cGAS (103), which exerts the protective effect in the development of atherosclerosis of *Apoe*^−/−^ mice (104). Suramin which competes with DNA for cGAS binding inhibited migration and proliferation of VSMCs induced by FAM3A overexpression or PDGF-AB treatment, leading to reduce the neointima hyperplasia (105–107). In addition, suramin prevents monocrotaline-induced pulmonary hypertension (108). Perillaldehyde which inhibit the binding of DNA to cGAS promotes perillaldehyde angiogenesis, which is beneficial in the treatment of ischemic cardiovascular diseases (109). In HFD-induced Apoe^−/−^ mice, Perillaldehyde prevented endothelial dysfunctions and increased NO generations, resulting in reducing the size of atherosclerotic plaque in aortic arteries (110). Competitive inhibitors in the catalytic site RU.521 has been reported to be effective in protection against septic cardiomyopathy induced by LPS (111, 112). Moreover, another competitive inhibitor in the catalytic site PF-06928215 negated palmitic acid (PA)-induced cardiomyocyte contractile dysfunction (113). Herein, It's been aggregated that reported cGAS inhibitors were proven to exert protective effects in cardiovascular diseases (Table 2).

The development of existing STING inhibitors focused mainly on the ligand-binding pocket and palmitoylation site using computer-aided design. Candidate molecules were then screened using high-throughput screening and their STING-inhibitory efficiency was verified in mice or humans. Inhibitors that target the ligand binding pockets bind to STING's endogenous ligand cGAMP, which prevents cGAMP from activating STING. Such inhibitors include SN-011, natural cyclic peptide Astin C, tetrahydroisoquinoline (compounds 1 and 18), etc. (114–116). Astin C improved PA-induced cardiomyocyte contractile dysfunction by inhibiting cGAS-STING pathway (113). The palmitoylation of STING sites Cys88 and Cys91 is necessary for the formation of polymeric complexes and the recruitment of downstream signaling pathway molecules during STING activation (8). Inhibitors that inhibit the Cys91 site include nitrofurans (C176, C178, C170, and C171), H151, and acrylamide (BPK-21 and BPK-25) (117). Among these, both C176 and H151 have protective effects on cardiomyopathy, including diabetic cardiomyopathy, myocardial infraction, and ischemia-reperfusion injury (17, 18, 34). Even more, two studies from different countries reported that C176 inhibits the progression of atherosclerosis induced by HFD or CKD in *Apoe*^−/−^ mice (21, 74). Nitro-fatty acids (NO_2_-Fas, NO_2_-cla, NO_2_-OA) have an inhibitory effect on both palmitoylation sites (118). Accumulated studies have reported that nitro-fatty acids showed the effective protection against cardiovascular diseases, which include ischemic ventricular arrhythmias, cardiac remodeling, abdominal aortic aneurysm, pulmonary hypertension, neointima formation, and endothelial injury (119–124). What's more, a safe and well-tolerated NO2-FAs, CXA-10, has been being investigated in phase II clinical trails for pulmonary hypertension (NCT04125745, NCT04053543, and NCT03449524). Herein, It's been aggregated that reported STING inhibitors were proven to exert protective effects against cardiovascular disease (Table 2). Therefore, the application of cGAS or STING inhibitors will provide a new strategy for the treatment of cardiovascular diseases.

## Conclusion and perspective

In the onset, progression and outcomes of cardiovascular diseases, the persistent inflammatory response is functionally important for contributing to adverse clinical outcomes (13–15). Yearly, the important role of cGAS-STING signaling pathway in sterile inflammation has historically been appreciated (11). Under pathological conditions, immune cells, vascular endothelial cells, VSMCs, or cardiomyocytes undergo mitochondrial damage or cell death, resulting in the leakage of mitochondrial or nuclear DNA into the cytoplasm (12). Stimulated by cytoplasmic DNA, the levels of inflammatory factors, chemokines, and IFNs are elevated through the cGAS-STING pathway (20, 27, 48, 125, 126). Herein, we made a conclusion that how cGAS-STING pathway is activated and how cGAS-STING pathway mediate sterile inflammatory cardiovascular disease. From this point, small molecule inhibitors targeting cGAS or STING may represent a novel approach for the treatment of sterile inflammatory cardiovascular diseases. Notwithstanding, in order to consider the clinical application of pharmacological inhibitors targeting cGAS or STING, the bioactivity, target selectivity, pharmaceutical absorption, and toxicity of these inhibitors need to be further identified. Moreover, based on the structure of these lead compounds, cGAS or STING inhibitors mentioned above, chemical optimization will benefit the clinical application of cGAS or STING inhibitors.

## Author contributions

YD, HZ, and LW drafted the manuscript text and prepared figures. SS drew the chemical structure. YD, XN, YH, WZ, and LW prepared the table. YQ, CH, and LW edited the paper. All authors contributed to the article and approved the submitted version.

## Funding

This study was funded by the National Natural Science Foundation of China (81670425).

## Conflict of interest

The authors declare that the research was conducted in the absence of any commercial or financial relationships that could be construed as a potential conflict of interest.

## Publisher's note

All claims expressed in this article are solely those of the authors and do not necessarily represent those of their affiliated organizations, or those of the publisher, the editors and the reviewers. Any product that may be evaluated in this article, or claim that may be made by its manufacturer, is not guaranteed or endorsed by the publisher.
